# Arachidonic Acid in Human Milk

**DOI:** 10.3390/nu12030626

**Published:** 2020-02-27

**Authors:** Norman Salem, Peter Van Dael

**Affiliations:** Nutrition Science & Advocacy, DSM Nutritional Products, Columbia, MD 21045, USA; peter.van-dael@dsm.com

**Keywords:** arachidonic acid, human milk, nutritional influences, lipid composition, eicosanoids, endocannabinoids

## Abstract

Breastfeeding is universally recommended as the optimal choice of infant feeding and consequently human milk has been extensively investigated to unravel its unique nutrient profile. The human milk lipid composition is unique and supplies specifically long-chain polyunsaturated fatty acids (LC-PUFAs), in particular, arachidonic acid (ARA, 20:4*n*–6) and docosahexaenoic acid (DHA, 22:6*n*–3). Arachidonic acid (ARA) is the most predominant long-chain polyunsaturated fatty acid in human milk, albeit at low concentrations as compared to other fatty acids. It occurs predominantly in the triglyceride form and to a lesser extent as milk fat globule membrane phospholipids. Human milk ARA levels are modulated by dietary intake as demonstrated by animal and human studies and consequently vary dependent on dietary habits among mothers and regions across the globe. ARA serves as a precursor to eicosanoids and endocannabinoids that also occur in human milk. A review of scientific and clinical studies reveals that ARA plays an important role in physiological development and its related functions during early life nutrition. Therefore, ARA is an important nutrient during infancy and childhood and, as such, appropriate attention is required regarding its nutritional status and presence in the infant diet. Data are emerging indicating considerable genetic variation in encoding for desaturases and other essential fatty acid metabolic enzymes that may influence the ARA level as well as other LC-PUFAs. Human milk from well-nourished mothers has adequate levels of both ARA and DHA to support nutritional and developmental needs of infants. In case breastfeeding is not possible and infant formula is being fed, experts recommend that both ARA and DHA are added at levels present in human milk.

## 1. Introduction

Optimal nutrition is critical in order to support adequate growth and development during infancy. Experts universally recommend breastfeeding as the optimal choice of infant feeding and consequently human milk has been extensively investigated to unravel its unique nutrient profile [1]. The lipid composition of human milk is complex. It has been estimated that it contains approximately one thousand different fatty acids. Human milk globally always contains the long-chain polyunsaturated fatty acids (LC-PUFAs) arachidonic acid (ARA, 20:4*n*–6) and docosahexaenoic acid (DHA, 22:6*n*–3) but at variable levels. This paper will review the lipid forms of fatty acids, particularly that of arachidonic acid (ARA, 20:4*n*6), in human milk as well as the relationship between human milk ARA levels and the influence of dietary factors. The latter is reviewed to establish the relationship between dietary factors and human milk levels in view of the totality of scientific evidence. The metabolic relationship between ARA, eicosanoids and other bioactive compounds as well as their presence in human milk will be presented. Finally, this paper will discuss recent data regarding the functionality and role of ARA during early life.

## 2. Lipid Composition and Lipid Forms of Human Milk

Human milk is a complex liquid specifically composed of a variety of carbohydrates, lipids, proteins and micronutrients and is designed to meet the nutritional needs of infants during the first year of life on its own. The lipids in human milk occur primarily in the physical form of milk fat globules or droplets. The lipid composition of human milk is influenced by many factors, including the stage of lactation, duration of feeding, gestational age at birth, diet and nutritional status as well as the physiological status of the mother [2]. Therefore, it is challenging to provide a specific level for the various lipids in human milk given the many variables influencing the level and variability of the lipid composition. In one study of over 2500 Danish mothers, the fat level ranged from 39 to 89 g/L [2]. Stated another way, total lipids comprise approximately 2–5% of the milk by weight. At day 7 of lactation, for example, total fat is 2.89%, with the following make up: phospholipids (0.8), cholesterol (0.7), triglycerides (98.5) [3].

The major classes of phospholipids (PL) in human milk are phosphatidylethanolamine (PE), phosphatidylcholine (PC) and sphingomyelin (SM), while smaller amounts of the acidic phospholipids phosphatidylserine (PS) and phosphatidylinositol (PI) are also present [3]. The quantitative distribution depends on various factors including stage of lactation. For example, mature human milk from mothers who gave birth at full term contains a concentration of various phospholipids (total 36.9) as follows: PE (29), PC (4.5), SM (3.3) (calculated as mg/100 mL of milk). Preterm human milk contains much more total PL (57.5) but colostrum contains more than double this amount (117) [4]. PE is the predominant phospholipid in human milk and its specific molecular forms have been determined using tandem-mass spectrometry [4]. The concentrations of the major molecular forms of PE in mature term-transitional milk and expressed in the units of mg/100 mL of milk were those containing 18:0, 18:1 (6.6); 16:0, 20:4 (5.3); 16:0, 18:0 (4.7); 18:0, 18:2 (4.2); 18:0, 20:4 (2.6). The docosahexaenoic acid (DHA, 22:6n3)-containing compounds also occurred at significant levels, with 16:0, 22:6 (1.6) and 18:0, 22:6 (3.1) predominating. Again, the preterm milk had much higher levels of these molecular forms of PE with approximately double the amounts of the ARA species and triple the amount of the DHA-containing compounds.

Triglycerides make up the major part of total fat (> 98.5%) in human milk. Detailed analysis of human milk triacylglycerides (TAGs) including the positional location of the various fatty acids has been performed as the structure has an influence on its metabolism [5]. The three major fatty acids of human milk-oleic acid (18:1), palmitic acid (16:0) and linoleic acid (LA, 18:2*n*6)-were preferentially located in a particular position on the TAG backbone, with oleic acid in the *sn*-1 position, palmitic acid in the *sn*-2 position and linoleic acid in the *sn*-3 position. The n-3 fatty acid, α-linolenic acid (ALA, 18:3n3), was similar to the LA in this respect. Both ARA and DHA were found more frequently in the *sn*-2 and *sn*-3 positions with ARA distributed equally between the two positions and DHA slightly more in the *sn*-2 position.

Human milk lipids are mainly found within the milk fat globules in human milk which are secreted by mammary epithelial cells. An early review by Stuart Patton, one of the pioneers of milk fat globule research, describes this secretory process [6]. TAGs appear to accumulate at focal points along the endoplasmic reticulum (ER) membrane and are released into the epithelial cell cytoplasm as droplets coated with ER membrane. These droplets then grow in volume most likely through fusion with each other creating cytoplasmic lipid droplets. The fat globules contain most of the milk fat, with the TAGs in the droplets’ core and glycerophospholipids as well as other polar lipids (e.g., glycolipids) on the membranous coating material. Fat globules mostly range from 1 to 8 µm in diameter, although there is also a small proportion of larger droplets. These droplets become enveloped in plasma membrane and have a large membranous surface area estimated at estimated at 23 ft^2^/2.1 m^2^ for one liter of mature human milk. These membranous milk structures are referred to as milk fat globule membranes (MFGM) and have been estimated to contain 2–6% of the mass of the globules. Keenan and Patton [6] estimated that the lipids of the MFGM preparations were predominantly triglycerides (58%), and approximately 22% phospholipids, 8% diglycerides and 7% unesterified fatty acids. The phospholipids in human milk are found predominantly within these MFGM and the main components are PE (37%), PC (30%), SM (26%), PI (5%) and PS (1%). The MFGM phospholipid distribution is very similar to that found in plasma membranes of mammary gland. Recently, infant formulas with MFG and MFGM have been placed on the market to mimic human milk lipid composition [7].

ARA is found in milk phospholipids in a higher percentage than in triglycerides although they occur at a much lower concentration. The mean human milk ARA and DHA levels reported for American mothers, measured at various stages of lactation between 26 and 40 weeks, living in the Washington DC area, were 0.55–0.60% and 0.21–0.24%, respectively [3]. ARA and DHA mature human milk levels reported for French mothers living in the port city of Marseille, which has fish readily available, were reported at 0.44% and 0.38% of total fatty acids, respectively [8]. Transitional human milk levels for the same mothers were higher levels for both ARA and DHA and were at 0.67% and 0.60%, respectively. Their colostrum ARA was slightly higher reaching 0.71%, whereas their DHA levels was lower at 0.47%. This study also provided data for the quantitative assessment of ARA and DHA in the phospholipid and triglyceride fractions, with approximately 90% of the ARA in the triglyceride fraction and the remainder in phospholipids for both transitional and mature milk. The data for DHA revealed that approximately 90% was found in the triglyceride fraction in transitional milk, but only 78% in mature milk. This was in spite of the higher percentages of both ARA and DHA in the phospholipid fraction relative to the triglyceride fraction of human milk. Therefore, it can be concluded that ARA and DHA in human milk are mainly located in the triglyceride fraction.

## 3. The Effect of Dietary Factors on Arachidonic Acid Levels in Human Milk

### 3.1. Variation across Countries

The ARA level of human milk varies widely across various countries and regions. One of the key factors that at least partially influences differences between countries is the diet. A 2006 review by Yuhas and colleagues compared the human milk fatty acid distribution across nine different countries [9]. The ARA levels were reported to range between 0.36% and 0.49%, with the lowest levels in the United Kingdom and the highest levels in China. The DHA levels ranged between 0.17% and 0.99%, with the lowest levels reported for both Canada and the United States and the highest levels in Japan. Higher levels, namely 0.74% of total fatty acids, were also reported for the Philippines.

A 2007 review by Brenna and colleagues surveyed worldwide ARA and DHA levels in human milk and included a total of 65 studies representing data for 2474 mothers [10]. They reported a mean ARA and DHA human milk level of 0.47% and 0.32% of total fatty acids, respectively. Of great interest were the differences reported between various countries. For ARA, the lowest levels (0.24%) were observed in France, whereas the highest levels (1.0%) were found in both Japan and rural South Africa. For DHA, the lowest value (0.06%) was reported for Pakistan, but similarly low values were observed in rural South Africa (0.1%) and Canada (0.12%), whereas the highest values were reported for the Canadian Arctic (1.4%) and Japan (1.1%).

In a 1999 comprehensive review, Jensen reported a range for ARA in what was termed “Western” women, from 0.05% to 0.87%, and in “Non-Western” mothers, 0.28–1.12% [2]. The values for Western women were comprised mainly of values from the US and Europe, whereas the non-Western values were mainly from the Asia Pacific area. For DHA, levels ranged from trace to 1.03% for Western mothers and from 0.00% to 2.78% in non-Western mothers. The reported trace levels and reports of absence of 0.00% are noted above but it is doubtful that the milk is completely devoid of DHA-rather, these values should be taken to indicate that they were below the level of sensitivity of the methodology or perhaps below the reporting level. Jensen summarizes a study of well-nourished Sudanese women and their breast milk fatty acid composition performed by Laryea [11]. They found an ARA content of approximately 0.5% and a DHA content of only approximately 0.05%. They report minimum and maximal values for both ARA and DHA. For ARA, values ranged from 0.28 to 0.86 while for DHA they ranged from 0.00% (undetectable) to 0.28%. Jensen also summarizes data from French women who also had a wide range for DHA values (0.00–0.82%) and ARA values (0.25–0.87%). Jensen summarizes data on moderately to severely malnourished Nigerian women with body mass indices of 20.2 and 16.4, respectively [12]. The ARA content was 0.60% in the moderately malnourished women but fell to undetectable levels in the severely malnourished women. DHA similarly fell as a function of malnourishment from a level of 0.30 to 0.10%. Thus, it is concluded that there is wide variation in both ARA and DHA levels with perhaps a wider range of DHA contents. It is suggested that the main source of this variation between countries and cultures is the difference in dietary intake of essential fatty acids. This contention is examined in controlled studies in the next section.

### 3.2. Dietary Intervention Studies

Literature data often report that *n*-3 fatty acids such as DHA are modulated by diet whereas *n*6 fatty acids like ARA are not. This section will review this general statement against the totality of available data for ARA and DHA in human milk with the aim to confirm or update as needed.

An early study published by Harris and colleagues in 1984 assessed human milk fatty acid composition upon daily consumption of 5 (for 28 days), 10 (for 14 days) or 47 (for 8 days) g of fish oil [13]. Analysis demonstrated a dose-dependent increase in human milk long-chain n-3 fatty acids with levels reaching 1%, 1.7% and 4.5% of total fatty acids, respectively. Fish oil consumption did not affect the human milk ARA level and remained at the baseline level of 0.4% of total fatty acids. A systematic review of 22 international studies by Amaral and colleagues studied the effect of dietary n-3 fatty acids on the human milk fatty acid profile [14]. They concluded that there was a dose-dependent increase in human milk *n*-3 fatty acids, principally DHA and in some cases also EPA, upon omega-3 fatty acid supplementation. The authors did not document the influence of omega-3 fatty acid supplementation on ARA content.

A very informative study regarding the effect of *n*-3 fatty acid supplementation on human milk fatty acid composition was performed by Makrides [15]. In this study, pregnant women received graded daily doses ranging between 0.2 and 1.3 g of DHA beginning on day 5 post-partum with the fatty acid profile of their breast milk examined at 12 weeks. The study reported a linear correlation between the increase in human milk DHA levels and the dietary DHA intake. At the highest DHA dose, human milk DHA levels increased up to 1.7% of total fatty acids. A similar study was performed by Helland and coworkers who supplemented pregnant women with large doses of cod liver oil [16]. Human milk DHA levels increased up to levels of 1.56% of total fatty acids. The human milk EPA levels were also increased, but the ARA levels remained unchanged. These studies clearly indicated that long-chain *n*-3 fatty acid dietary intake during pregnancy directly correlates with their human milk long-chain omega-3 fatty acid levels.

Fewer studies have assessed the effect of variable ARA dietary intake or supplementation on the fatty acid content of human or mammalian milk. Hadley and colleagues studied the effect of rat dams fed a diet with DHA only (0.32 wt%), ARA only (0.6 wt%), ARA:DHA at a 1:1 ratio and ARA:DHA at a 2:1 ratio on the fatty acid composition of the dam’s milk collected from the stomach contents of the pup [17]. The ARA level was at 1.8% with supplemented DHA only, increased marginally up to 2.0% when an equal amount of ARA was added to the diet, but increased significantly up to 2.8% of total fatty acids when the diet contained a 2:1 ARA:DHA ratio. These animal data clearly indicate that maternal dietary intake of ARA increases the ARA milk levels. It is also important to emphasize that the DHA level in the dam milk was not altered in this case by increasing the dietary ARA intake.

A randomized, controlled human trial studied the effect of a varying daily supplemental ARA intake (0, 200 or 400 mg) provided to lactating mothers in combination with long-chain *n*-3 PUFAs (320 mg DHA and 80 mg EPA) compared with no supplemental long-chain PUFAs on the human milk total lipid fatty acid composition over an 8 week period [18]. At the end of the 8 week intervention period, the total human milk ARA levels increased with increasing ARA supplementation from 0.4% (no ARA), to 0.49% (200 mg/day of ARA) and up to 0.56% of fatty acids (400 mg/day of ARA). There were no significant changes in the human milk DHA levels over the 8 week intervention period for any of the groups. Therefore, the authors concluded that there was a significant and dose-dependent increase in human milk ARA levels in response to increasing maternal ARA dietary intake. There was also a strong and positive correlation of the human milk ARA and DHA levels with the maternal erythrocyte phospholipid levels. A similar study assessed the effect of a short, 7 day intervention with 300 mg/day of supplemental ARA, alone or combined with EPA (100 mg) and DHA (400 mg), on the breast milk ARA level of lactating mothers. The data found no significant increase in human milk ARA levels, but a trend towards decreased long-chain *n*-3 fatty acids levels was observed [19]. However, these authors observed that when the ARA supplemental dose was given together with EPA/DHA there tended to be an increase in milk ARA and long-chain *n*-3 PUFAs levels. It must be noted that the short, one-week duration of the ARA supplementation most likely influenced the outcome of the study and the lack of human milk ARA responsiveness.

Another human study investigated the dietary and metabolic pathways of human milk ARA using stable isotope labelled linoleic acid (^13^C-LA) and preformed ARA in lactating women [20]. The estimated transfers of LA and ARA from diet into human milk was approximately 33% and 12%, respectively. The percentage of de novo synthesized ARA from its precursor LA in human milk was found to be very low and only approximately 1% of the total amount of ARA secreted in the human milk. Thus, the origin of the human milk ARA content is primarily from preformed dietary ARA.

In spite of the scientific evidence, it is often stated that the human milk ARA levels are relatively stable and not responsive to dietary intake. Generally, this statement is supported by the quoted lack of correlation between dietary ARA intake in a population and the human milk ARA levels. For example, one study compared the fat and fatty acid composition of vegetarians and omnivores in American mothers in 1985 [21]. They found that the human milk ARA levels in vegetarians and omnivores was 0.30% and 0.28%, respectively, and for DHA, the values were 0.05% and 0.06%. Dietary records for these mothers indicated that the omnivores ate 50 g/day of animal protein while the vegetarians reported 20 g/day; animal fat consumption was 47 g/day for omnivores and 25 g/day for vegetarians. Since ARA is found in the meat and animal fat, it follows that the omnivores had a considerably higher intake of ARA than did the vegetarians, but this produced no change in the milk ARA content. Note that the same argument could be applied to the DHA in this case.

In any case, how might these data showing little or no response to dietary input of ARA in milk fatty acids be reconciled with the ARA supplementation studies noted above? The answer lies, at least in part, in the disparity in *n*-6 and *n*-3 fatty acid intake and the resultant body burden of these fatty acids. A recent global survey of lipid intake found that the global mean for *n*-6 fatty acid intake (predominantly LA) was 5.9 en% (energy%) while intake of plant-based *n*-3 fatty acids (mainly ALA) had a global mean of only 1.37 g/day with seafood-based *n*-3 intake of 163 mg/day [22]. The US mean intake of *n*-6 fatty acids is 6.7 en% with very high values in Western (5.2), Eastern (8.0) and Central (7.9) Europe, East Asia (8.5) and tropical Latin America (6.9). Lower values for *n*-6 intake are found in much of Africa and Southeast Asia. The competition for incorporation into membrane lipids of the *n*-6 and *n*-3 fatty acids has long been appreciated and quantitative equations have been developed [23]. In Westerners, and increasingly in the whole world, there is a preponderance of *n*-6 fatty acids in our bodies [24] due mainly to the widespread use of seed oils, i.e., soybean, corn and safflower oils [25]. The enzymes that metabolize LA and ARA are partially or completely saturated and thus minor changes in LA or ARA intake have a diminished effect [26]. When larger doses of ARA are used in an intervention study for a reasonably long period, however, this may be sufficient to alter the tissue lipid profile. The preponderance of *n*-6 fatty acids in our tissues may explain, in part, why Western women have up to twice as much ARA relative to DHA in their milk [2].

## 4. Bioactive Metabolites of Arachidonic Acid in Human Milk

### Eicosanoids

Eicosanoids are a group of compounds formed from ARA and possess high biological activity in many organ systems. They may also be formed by enzymes such as cyclooxygenase, lipoxygenase or cytochrome P450 from ARA as well as other polyunsaturated fatty acids (PUFAs) that are, similar to arachidonic acid. They function in diverse physiological systems and pathological processes, including, among others, the modulation of inflammation. In performing these roles, eicosanoids most often act as autocrine signaling agents to impact their cells of origin or as paracrine signaling agents to impact cells in the proximity of their cells of origin. Eicosanoids may also act as endocrine agents to control the function of distant cells. Their presence in human milk was confirmed not too long after the discovery of prostaglandins, a group of eicosanoids made by the action of cyclooxygenases [27], but their function there is still uncertain. The prostaglandin (PG) biomediators produced from arachidonic acid that are present in human milk include the cyclooxygenase products PGE_2_ and PGF_2α_, the PGF_2α_ metabolite 13,14-dihydro-15-keto-PGF_2α_, (DHKPGF_2α_,), the prostacyclin inactivation product 6-keto-PGF_1α_, and the thromboxane (TX) A_2_ inactivation product TXB_2_ [28,29,30]. Eicosanoids produced from arachidonic acid by the lipoxygenase and cytochrome P_450_ pathways also are present in human milk [28,30,31]. The lipoxygenase products present are lipoxin (LX) A_4_ and LXB_4_, leukotriene B_4_ (LTB_4_), the hydroxy-eicosatetraenoic acid (HETE) regioisomers 5-HETE, 8-HETE, 9-HETE, 11-HETE, 12-HETE and 15-HETE, and the oxidized HETE metabolites 5-oxo-ETE and 15-oxo-ETE. The products formed by the cytochrome P_450_ pathway are 20-HETE, the epoxy-eicosatrienoic acid (EET) regioisomers 11,12-EET and 14,15-EET, and the EET metabolites 8,9-dihydroxyeicosatrienoic acid (DHET), 11,12-DHET and 14,15-DHET.

Current data regarding eicosanoids in human milk mainly focused on the prostaglandins PGE_2_ and PGF_2α_. These are present in human milk but not in infant formulas prepared from bovine milk [26]. Levels reported for PGE_2_ and PGF_2α_ in human milk in different studies vary widely [28,32,33,34], which is primarily due to methodological differences [28,29].

Data for PGE_2_ and PGF_2α_ levels in human milk from mothers who delivered term or preterm infants did not differ significantly [27,32,33], neither between milk collected at different times of lactation [27,32,33,34] or between foremilk and hindmilk [27,35]. Similarly, no significant differences were reported in the human milk levels for the lipoxygenase and cytochrome P_450_ products relative to the time of lactation [30,31].

PGE_2_ is synthesized in the mammary gland by the combined action of cyclooxygenase-1 and the microsomal prostaglandin E_2_ synthase-1, and the amount produced increases with development of the gland [36]. Macrophages contained in the milk also synthesize PGE_2_ [37,38,39]. The amount formed is stimulated when the macrophages are activated by endotoxin or zymosan [38], and it is inhibited by lactoferrin, an iron-binding protein with antibacterial properties that is rich in colostrum [38]. However, no correlation has been observed between the PGE_2_ and ARA levels in milk obtained from mothers who delivered either term or preterm infants [40]. Thus, it appears that macrophage PGE_2_ production is not dependent on the ARA level available in human milk. Likewise, the human milk PGE_2_ and PGF_2α_ levels were not affected by the presence in the maternal diet of hydrogenated or non-hydrogenated fats [41].

The gastrointestinal tract contains prostaglandin receptors [42], and prostaglandins have many functional effects in the stomach and intestine [43,44,45]. Furthermore, only minimal degradation occurs when PGE_2_ and PGF_2α_ are incubated in vitro with human milk or gastric juice obtained from infants [46], indicating that these prostaglandins are likely to have a high degree of stability when they are ingested by the infant [46]. These findings suggest that the prostaglandins ingested in human milk may have cytoprotective actions or beneficial effects on intestinal motility and intestinal transport [32,35,39,41,46]. This may be one advantage of human milk over infant formulas derived from bovine milk [27]. However, careful measurements of eicosanoids in human milk by RIA following HPLC purification or by LC–MS/MS indicate that the concentrations are much lower than previously reported [29,30,33], perhaps too low to exert such biological effects [29]. Therefore, the functional significance of the eicosanoids synthesized from arachidonic acid that are present in human milk is uncertain and an issue for future research [33].

## 5. Endocannabinoids and Endocannabinoid-Like Metabolites in Human Milk

The term endocannabinoid refers to the following nine fatty acid-derived endogenous ligands of the cannabinoid receptors, namely, anandamide (anandamide, AEA), 2-arachidonylglycerol (2-AG), *N*-arachidonyldopamine (NADA), noladin ether, virodhamine, *N*-dihomo-gammalinoenoylethanolamine, *N*-docosatetraenoylethanolamine and oleamide [47]. ARA-derived AEA and 2-AG and their receptors are abundantly present in humans from the embryonic stage onwards and as early as 14 weeks of gestation [48]. This early life presence suggests the importance of the endocannabinoid system in development. In animal models, activation of the endocannabinoid receptor CB1 was shown to be critical for the initiation of the milk sucking response which is necessary for growth and survival during the early stages of life [49]. Both AEA and 2-AG have been detected in human milk [28]. However, a wide range of concentrations has been reported for AEA and 2-AG, ranging from the lowest levels at 0.06 and 7.3 nM [28] to the highest levels at 8.6 and 890 nM, respectively [50]. The variability is presumably due to varying storage conditions [49] and/or analysis methods [51]. Independent studies have, however, consistently reported human milk levels for 2-AG to be at least 100-fold higher as compared to AEA levels [28,50,52].

Human milk also contains other endocannabinoid-like *N*-acylethanolamines (NAE) such as *N*-stearoylanolamine (SEA), *N*-palmitoylamine (PEA), *N*-oleoylethanolamine (OEA), *N*-eicosapentaenoylethanolamine (EPEA) and *N*-docosahexaenoylethanolamine (synaptamide, DHEA). The reported concentrations of these compounds are generally within a similar range to that of AEA (0.04-9 nM) [28,52,53,54]. The metabolic functions of these compounds are important during early life: OEA, for example, induces satiety, inhibits food intake, and increases energy catabolism [55,56,57]. Synaptamide, an endogenous metabolite of DHA, targets GPR110 (ADGRF1) and potently promotes neurogenesis, neurite growth and synaptogenesis in developing brains [58] and inhibits inflammatory responses [59,60].

The NAEs and 2-AG are subjected to enzymatic degradation by fatty acid amide hydrolase (FAAH) and by monoacylglycerol lipase, respectively, in the gastrointestinal (GI) tract and therefore, their delivery into the blood stream may not be efficient. However, these compounds may act in the gut before degradation, although the presence of endocannabinoid receptors in the GI system has not been clearly demonstrated except in inflammatory conditions [61]. A significant correlation was observed between the level of synaptamide and the precursor DHA [53], suggesting that maternal supplementation of DHA can raise the synaptamide level in human milk. Likewise, the levels of other NAEs in human milk can be manipulated by the maternal intake of corresponding fatty acid-rich supplements or diet. Based on the physiologic significance of the endocannabinoid system and endocannabinoid-like compounds, the presence of these NAEs in the human milk may have considerable influence on the health and development of breastfed infants.

## 6. Advances in Arachidonic Acid Function: Infant Requirements

The eicosanoids and endocannabinoids produced from ARA have many and varied functions in the human body. ARA is as essential as is DHA for the development and functioning of the nervous system [62,63]. ARA is rapidly accumulated in the fetal brain during early development and reaches a very high concentration similar to that of DHA [64,65]. Like DHA, ARA is present mainly in membrane phospholipids. Breastfeeding has been associated with optimal brain development and function [66]. It has been associated with the unique lipid composition of human milk and, in particular, the presence of ARA and DHA [67]. Consequently, the role of ARA and DHA during early life has been extensively studied, due in part to the ARA and DHA supplementation of infant formulas to mimic the composition of human milk [67]. Many infant feeding studies assessed the effect of ARA and DHA-supplemented infant formulas on brain development and cognitive functions and reported beneficial effects for supplemented as compared to non-supplemented infant formulas. Improved functional outcomes in formula-fed infants were reported for a combination of ARA and DHA at a 2:1 ratio [68]. Although the positive effect of LC-PUFA supplemented infant formula has often been attributed to DHA, it is almost certainly due to a combination of both DHA and ARA similar to the outcomes found for breastfed infants, who always receive both LC-PUFA through human milk [69]. Moreover, in a few studies, it has been demonstrated that the addition of ARA to a DHA-containing formula imparts a benefit or an increased benefit. For example, supplementation of term infants with ARA + DHA resulted in a significant 7-point increase in cognitive functionality measured as Intelligence Quotient (IQ) using the widely used Bayley’s Mental Development Index relative to a vegetable oil-based control formula [70]. The importance of this finding is that while the DHA-supplemented formula resulted in a 4-point increase in the mean score, it did not reach statistical significance, demonstrating the clear combined benefit of the presence of both ARA and DHA in the infant’s diet during early development. Both DHA only and combined ARA and DHA-supplemented formulas resulted in functional benefits for cognitive and motor subscales of the Bayley’s Scales of Infant Development (BSID-II).

Alshweki et al. reported that preterm infants receiving a combined ARA and DHA-supplemented preterm infant formula at a 2:1 ratio scored 9 points higher in the Brunet Lezine test for psychomotor development at 24 months of age as compared to preterm infants receiving a similar formula but only supplemented at a 1:1 ratio [71]. At 12 months of age, the infant plasma fatty acyl distribution demonstrated significantly higher ARA levels. DHA levels on the contrary were not significantly increased. Support for a higher ARA to DHA ratio at 2:1 comes also from the DIAMOND study. In this study, term infants received infant formulas with only vegetable oils or supplemented in addition with increasing DHA levels (0.32%, 0.66% and 0.96%) at a constant ARA level at 0.66% [72]. When combining data for all infants receiving the supplemented infant formulas, performance was found better on most behavioral tasks as compared to the infants receiving unsupplemented infant formula. Of interest for the present discussion was the observation that in several brain development measures the infants receiving the 0.66% DHA-supplemented formula performed better than those receiving the higher 0.96% DHA-supplemented formula during later development. For example, children receiving the 0.66% DHA-supplemented formula demonstrated improved outcomes on several behavioral tasks, namely the Peabody Picture Vocabulary Test, the Dimensional Change Card Sort and measures of sustained attention, as compared to the infants receiving the 0.96% DHA-supplemented formula. The only exception was the outcomes of the Stroop test in which results for both 0.66% and 0.96% DHA-supplemented formulas were similar and better as compared to the unsupplemented formula. In a subsequent study, Columbo and colleagues reported that the erythrocyte fatty acid composition of the infants ARA showed an inverted U shape and fell off at the high formula DHA level [73]. They suggested that an imbalance in ARA intake may lead to a decrease in the infant or child performance in these behavioral tasks. This hypothesis is supported by results of a study of full-term baboon neonates consuming the same ratios of ARA:DHA as in the DIAMOND study [74]. The data from this study demonstrated increasing DHA content in brain as well as other tissues in the infants as DHA in the formula increased; however, at the higher DHA intake, the ARA content in two brain areas, the globus pallidus and superior colliculus was decreased. This study supports the observation in the DIAMOND study that an adequate ARA and DHA supplementation and ratio are likely critical to ensure a balance in ARA and DHA brain levels.

One reason why the specific functions of ARA during early mammalian development have not been as carefully detailed as for DHA relates to the fact that there has not been an adequate animal model for it to be studied. When n-6-deficient diets were given to rodents, for example, their growth and brain size were severely impaired, so that the results could not be well ascribed to ARA deficiency. Often a fat-free diet was used, which may have led to decreased fat reserves for energy balance [75]. More recently though, a delta-6 desaturase knockout mouse (D6D KO) was developed by Nakamura and coworkers [76], enabling animals to be fed LA and *n*-3 PUFAs and the animals still had no ARA except what they were born with. Moriguchi and colleagues combined this animal model with their unique ability to artificially rear mice from the first days of life on an artificial milk wherein the fatty acyl composition can be completely controlled [77]. This model was used in an elegant experiment by Moriguchi and colleagues to study the metabolic effects of DHA alone, ARA alone and both combined. When the D6D KO mice were given an artificial milk with only ARA added, the animals were hyperexcited in activity tests and their motor coordination was worse than the wild type [78]. The combination of both ARA and DHA, but not DHA only, was able to correct both adverse effects. In a subsequent experiment, it was demonstrated that ARA-deficient mice had a decrease in body and brain weight which could be corrected by addition of ARA alone or both ARA and DHA [79]. Moreover, the D6D KO mice had deficits in learning and memory-related performance (Morris Water Maze) which could be corrected by DHA or DHA +ARA but not by ARA alone or ARA+EPA. These recent studies clearly show that both ARA and DHA are essential fatty acids for mammalian growth and development and that only a combination of ARA and DHA can provide for optimal neural development.

It may be argued that this level of ARA deficiency may not be encountered in human infants. However, a case of human delta-6-desaturase (D6D) deficiency has been noted [80]. Although this may be rare and the overall incidence is unclear, the occurrence of single nucleotide polymorphisms (SNPs) with desaturase variants that lead to lower levels of PUFA metabolism and content have now been described [81]. Variations in desaturase genes have been described across ethnic groups and countries [82]. In German adults, for example, strong associations were found between variants in D6D and D5D genes and the phospholipid fatty acid composition [83]. Variants led to higher n-6 PUFA precursors (linoleic acid) and lower end products like ARA. For this discussion, it was most important that the authors noted: “*The most significant associations and the highest proportion of genetically explained variability (28%) were found for arachidonic acid (C20:4n-6)*.” The minor alleles had a fairly significant frequency; for example, the rs174583polymorphism in the FADS2 gene had a minor allele (2/2) frequency of 13% and several others were 11–16%. Barman et al. found similar reductions in ARA and increases in its precursor in young children with FADS variants and noted that similar albeit smaller changes were observed when the children were 13 years of age [84]. These SNPs also alter breast milk fatty acid composition, particularly ARA [85]. Salas Lorenzo and colleagues recently studied the effect of ARA and DHA-supplemented infant formulas on fatty acid levels in infants with different FADS genotypes [86]. They concluded that the infant FADS genotype could contribute to narrow the gap of ARA and DHA concentrations between breastfed and formula fed infants. They further recommended that when infants are not breastfed, combined ARA and DHA-supplemented formulas should be considered since they provide better ARA and DHA concentrations compared to infants receiving non-supplemented infant formulas.

As SNPs differ among genetically different populations, it is critical to assess ARA and DHA dietary needs in light of their genetic polymorphism. Tanjung and colleagues [87] reported the similarity of the FADS1–3 SNP distribution patterns in an Indonesian and a previously studied Mexican population [88] are noteworthy, as both differed from European populations. Both the Indonesian and the Mexican populations showed the minor alleles to be associated with high levels of LC-PUFA synthesis, whereas the major genotypes were associated with low levels of LC-PUFA synthesis. Therefore, the supply of preformed LC-PUFA may be particularly important in these populations to achieve a similar plasma content.

Most recently, a position paper of the European Academy of Paediatrics and the Child Health Foundation was published recommending that infant and follow-on formula should provide both ARA and DHA [69]. The authors, an international group of experts in infant and lipid nutrition, concluded that DHA should equal at least the mean content in human milk globally (0.3% of fatty acids) but preferably reach 0.5%. As to ARA, they strongly recommend that it should be provided along with DHA, even though optimal ARA intake amounts remain to be defined. At amounts of DHA in infant formula up to ∼0.64%, ARA content should at least equal the DHA content. Finally, they recommended that further well-designed clinical studies should evaluate the optimal intakes of ARA and DHA in infants at different ages based on relevant outcomes.

## 7. Conclusions

Human milk contains relatively small but important amounts of the highly unsaturated fatty acids ARA and DHA primarily in the form of trigylcerides and secondarily in phospholipids, principally phosphatidylethanolamine, and contained in the milk fat globules. Dietary factors are important in determining human milk ARA content just as it is for DHA; this is suggested by the wide variations across countries and cultures and is made clear by direct animal and human interventional studies where ARA dietary intake is varied. The main source of human milk ARA is preformed ARA rather than originating via the metabolic pathway of linoleic acid elongation/desaturation. A variety of bioactive molecules are present in human milk formed from ARA as their precursor including prostaglandins, hydroxylated fatty acids, as well as anandamide and a variety of related endocannabinoids. The prostanoids may provide cytoprotective benefits for the infants’ gut and endocannabinoids may play a role in initiation of the milk suckling response, modulation of satiety and energy metabolism and influence brain development thru their effects on neurite outgrowth and synaptogenesis.

ARA rapidly accumulates in the fetal and neonate brain phospholipids just as does DHA. Clinical trials investigating LC-PUFA supplementation have generally shown benefits for neural and retinal development when both ARA and DHA have been given, most often in a 2:1 ratio. Recent mechanistic work has demonstrated clearly that ARA is an essential fatty acid required for body and brain growth as well as optimal neural function. The capacity of infants to form ARA endogenously from its dietary precursors has been shown to be quite variable in significant segments of the population and across countries due to single nucleotide polymorphisms in the desaturase enzymes involved. Such variations in metabolic capacity may create or exacerbate deficiencies in ARA that can best be overcome by assuring that infants receive preformed ARA in their diets along with balanced levels of DHA.
